# Exploring Exercise Intervention as a Therapeutic Catalyst within the Mental Health and Addiction Program in Nova Scotia: A Proof-of-Concept Study

**DOI:** 10.1192/j.eurpsy.2024.1724

**Published:** 2024-08-27

**Authors:** S. Obeng Nkrumah, R. da Luz Dias, E. Eboreime, B. Agyapong, S. Sridharan, V. I. O. Agyapong

**Affiliations:** ^1^Department of psychiatry, Nova Scotia Health; ^2^Department of psychiatry, Dalhousie University, Halifax, NS; ^3^Department of psychiatry, University of Alberta, Edmonton, AB, Canada

## Abstract

**Introduction:**

Many mental health conditions, including anxiety, mood disorders, and depression, can be effectively treated at a relatively low cost. Exercise interventions can be a therapeutic strategy, but even though exercise has consistently been shown to improve physical health, cognitive function, and psychological well-being, as well as reduce depression and anxiety symptoms, this intervention is often neglected in mental health care services.

**Objectives:**

The study aims to assess the feasibility of incorporating an Exercise Intervention Program (EIP) as a therapeutic pathway within the Mental Health and Addictions Program (MHAP) in Nova Scotia, as well as to evaluate the effectiveness of the program on mental health outcomes and incremental costs, and the patient acceptability and satisfaction with the program.

**Methods:**

This proof-of-concept study has a pragmatic, prospective, controlled observational design with an embedded one-phase qualitative component. Patients with a primary diagnosis of depression or anxiety attending the Rapid Assessment and Stabilization Program (RASP, Halifax, Nova Scotia, Canada) will be offered to receive 60-minute exercise sessions three times per week, per 12 weeks. Patients with similar mental health conditions that have opted to wait for Cognitive Behavioral Therapy (CBT) with the community provider and declined from the EIP will be part of the control group. A certified recreational therapist will conduct the EIP. Participants of both groups (EIP and control condition) will be assessed at baseline and then weekly for four weeks, six weeks and then at 12 weeks post-enrollment. Primary outcomes include differences in the mean change in functional (well-being, resilience, and recovery) and symptom variables (depression, anxiety, and suicidal risk), which will be assessed through online validated scales/questionnaires. Service variables (patient acceptance and satisfaction) and health care utilization (crisis calls, emergency department visits, hospital admissions and readmissions, length of stay for each admission) will comprise the secondary outcomes.

**Results:**

The results of the study will provide information about the effectiveness of EIP in the treatment of anxiety and depression compared to those only wait-listed to receive CBT or counselling from a CMHA provider. The study will also inform about the acceptability and satisfaction of the EIP, as well as the incremental cost-effectiveness of the intervention compared to the control condition.

**Conclusions:**

This proof-of-concept study will demonstrate the effectiveness of EIP as an adjunctive or alternative therapeutic option for the treatment of anxiety and depression in patients seeking mental health support from the MHAP in Nova Scotia.

**Disclosure of Interest:**

None Declared

